# Probiotics for the Prevention of Antibiotic-Associated Diarrhea

**DOI:** 10.3390/healthcare10081450

**Published:** 2022-08-02

**Authors:** Kira Kopacz, Sangita Phadtare

**Affiliations:** Cooper Medical School, Rowan University, Camden, NJ 08103, USA; kopacz36@rowan.edu

**Keywords:** probiotics, antibiotic-associated diarrhea (AAD), microbiome

## Abstract

Several communities have started using probiotic-rich fermented foods as therapeutic options with presumed medicinal powers. We now know the importance of microbiome balance and how probiotics can restore imbalances in the microbiome. Probiotics have been tested for a number of clinical uses such as the prevention of antibiotic-associated diarrhea (AAD), the treatment of various diseases such as *H. pylori* infection, irritable bowel disease, vaginitis, the prevention of allergies, and necrotizing enterocolitis in newborns. AAD has been the most indicated therapeutic use for probiotics. AAD is a common side effect of antibiotic usage, which affects up to 30% of patients. The hypothesis behind using probiotics for AAD is that they help normalize an unbalanced flora. There are many potential mechanisms by which probiotics support intestinal health such as (i) boosting immunity, (ii) increasing gut barrier integrity, (iii) producing antimicrobial substances, (iv) modulating the gut microbiome, (v) increasing water absorption, and (vi) decreasing opportunistic pathogens. Many randomized-controlled trials including the strain-specific trials that use *Lactobacillus* and *Saccharomyces* and meta-analyses have shown the benefits of probiotics in addressing AAD. Although adverse events have been reported for probiotics, these are broadly considered to be a safe and inexpensive preventative treatment option for AAD and other gastrointestinal disorders.

## 1. Introduction

The global probiotic market is worth approximately $15 billion USD per year and is growing at an estimated annual rate of 7% [1]. With the ever-growing interest in probiotics with respect to human health, understanding both its historical context and its current uses is important for future implications in medicine. The word probiotic translates from the Latin “pro” and Greek “bios” to mean “for life” and was originally coined by the German scientist Werner Kollath in 1953 [2,3,4]. Many societies have been using probiotic-rich fermented foods as therapeutic options. Recently, great strides have been made to understand the human gut microbiome and how microbiome imbalances (dysbiosis) lead to several disease conditions. Probiotics are thus attractive as a health-promoting approach to restore the normal intestinal environment [5]. One of the most indicated therapeutic uses for probiotics is the prevention of antibiotic-associated diarrhea (AAD).

## 2. History of Probiotics

In the 1800s, scientists wrote about the apparent health benefits of ingesting fermented milk products [6]. However, the mechanism of action leading to these benefits remained unclear. Louis Pasteur successfully identified the yeast and bacteria responsible for fermentation but excluded their link to any health effects [7]. Then in 1905, Elie Metchinikoff, a Russian scientist who had worked with Pasteur, made an association of the longevity among Bulgarians not simply to the yogurt they often consumed, but to the *Lactobacilli* used to ferment it and their presence in the colon [8]. Subsequently, Henry Tissier, in 1906, isolated *Bifidobacterium* from an infant and suggested that it had the potential to displace pathogenic bacteria in the gut [9]. These hallmark discoveries catalyzed research into health-promoting and disease-fighting microbes in the next century. In 2001, an Expert Panel was created at the request of the Food and Agriculture Organization of the United Nations, and backed by the World Health Organization, to define probiotics as: “Live microorganisms which when administered in adequate amounts confer a health benefit on the host” [10]. The first available probiotics were single-species-based, for example, those belonging to the *Saccharomyces* or *Lactobacillus* genera. This was followed by the creation of probiotics that included a wider variety and a larger number of microorganisms (e.g., 10^8^ to >10^10^ organisms).

## 3. Clinical Uses for Probiotics

**Figure 1:**
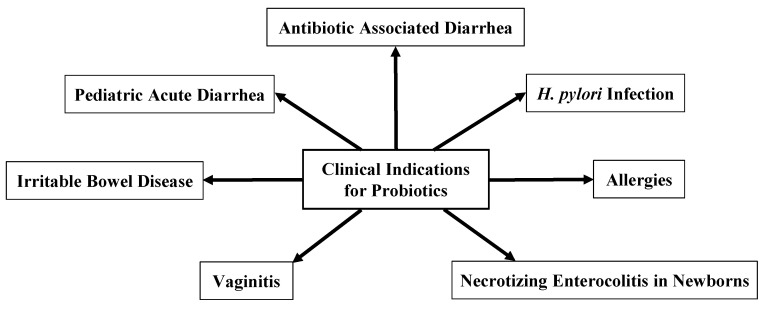
Clinical indications for probiotics.

The microbiome is a complex ecosystem that is integral to many aspects of human health including energy metabolism [11], neurotransmitter production [12], vitamin absorption [13], and protection against pathogens [14]. Any alteration to this complex network can tilt the scales and cause adverse health outcomes in humans. One therapeutic option to replace and rebalance the flora is to administer probiotics [15]. Probiotics have been tested for a number of clinical uses. The most common clinical use of probiotics is the prevention of antibiotic-associated diarrhea. Other common indications include the treatment of *H. pylori* infection [16,17], the treatment of pediatric acute diarrhea, the prevention of allergies [18], the treatment of irritable bowel disease, the treatment of vaginitis [19], and the prevention of necrotizing enterocolitis in newborns [20,21] (Figure 1). However, the findings of probiotics’ clinical efficacy vary by probiotic strain and by indication.

## 4. Antibiotic-Associated Diarrhea

Probiotic research for the treatment of AAD is growing due to the increasing prevalence of AAD and the subsequent strain on the healthcare system. AAD is a common side effect of antibiotic usage that affects up to one-third of patients who are treated with antibiotics [22]. There are different mechanisms and factors by which antibiotics can not only cause but also increase diarrhea, as follows:

**(i)**  **Altering the diversity of gut bacteria:** While antibiotics kill and target pathogens, they also impact the symbiotic bacteria integral to the gut microbiome. This decrease in bacterial diversity in the GI tract can drastically alter the immunological ecosystem. This puts the patient at greater risk of opportunistic infections and allows pathogens to competitively outcompete other bacteria [23,24,25].
**(ii)**  **Age of patient:** While AAD can occur in any patient population, the pediatric population is particularly at risk. Since the infant microbiome is not fully developed, antibiotic use in this population can cause a longer, more drastic effect on the microbiome, including an increase in *Proteobacteria* and a decrease in the diversity of *Actinobacteria* [24].
**(iii)**  **Spectrum of antibiotics:** Antibiotic characteristics such as their mechanism of action, pharmacokinetics, and dosage can not only affect the targeted pathogen but can also lead to unintended consequences such as AAD. Broad-spectrum antibiotics such as clindamycin, which are particularly active against anaerobes, are associated with higher rates of AAD. On the other hand, narrow-spectrum antibiotics typically produce lower rates of AAD [26,27].
**(iv)**  **Metabolic disturbances:** The gut microbiome plays an important role in nutrition and metabolism. While most carbohydrates are absorbed in the small intestine, some carbohydrates are fermented by the bacteria and turned into short-chain fatty acids (SCFAs). When antibiotics kill and lyse these bacteria, excess amounts of non-absorbable carbohydrates remain in the gut. These non-absorbable carbohydrates pull in water by osmosis as they move towards the large intestine. This leads to the development of osmotic diarrhea [27].
**(v)**  **Loss of colonization resistance:** Colonization resistance is the ability of bacteria to prevent pathogenic microbes from invading. The gut microbiome regulates many metabolites including bile acids, carbohydrates, and amino acids. These metabolites help to defend against pathogens. One example is the regulation of *Clostridium difficile* through secondary bile acids. Secondary bile acids are produced by gut bacteria and inhibit *C. difficile* growth. Antibiotics destroy the gut microbiome, leading to the diminishment of secondary bile acids. This then allows *C. difficile* to flourish [27].

AAD occurs in patients from the start of the treatment and can last up to two months after the end of the treatment [28]. For most clinicians, diarrhea is defined as three or more watery or loose bowel movements per day for at least two consecutive days. While patients can be given antibiotics in both inpatient and outpatient settings, most antibiotics are prescribed by primary care physicians [29]. Studies have shown that a significant bulk of antibiotic use, especially in outpatient settings, may be inappropriate. Awareness is now being raised to reduce antibiotic over-prescription. However, when antibiotic therapy is necessary, it is useful to have an effective, accessible, and safe method to prevent unwanted side effects such as AAD [30]. Recently, probiotics have become a more common option for patients suffering from AAD as a safe way to reduce the adverse side effects of antibiotics on gastrointestinal function [31].

## 5. Probiotics in Preventing AAD

**Figure 2:**
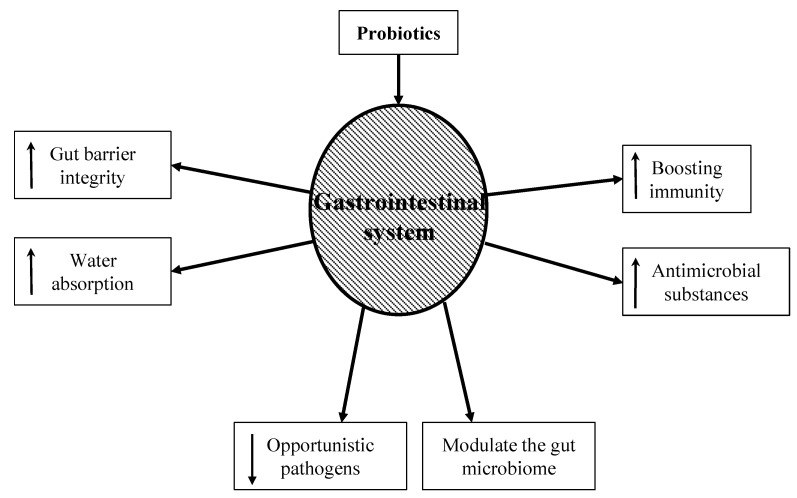
Hypothesized role of probiotics in the treatment of AAD. Upward and downward arrows inside the word boxes indicate increase and decrease, respectively.

Probiotics are a vast market filled with various strains with unspecified benefits. Many probiotic strains have been tested. The *Lactobacillus* genus, *Saccharomyces* genus, and *Bifidobacterium* genus are the most studied for their use as probiotics [32]. The hypothesis behind using probiotics to mitigate the symptoms and pathophysiology of gastrointestinal disorders is that they can help normalize an unbalanced flora [33]. Figure 2 shows potential mechanisms by which probiotics can support intestinal health. These mechanisms include:

**(i)**  **Boosting immunity:** While the exact mechanism is still unknown, probiotic bacteria have been shown to boost the humoral immune response by increasing the numbers of IgM-, IgG-, and IgA-secreting cells. They also stimulate nonspecific immune responses such as activating macrophages [34].
**(ii)**  **Increasing gut barrier integrity:** The intestinal barrier is a heterogeneous system composed of a mucus layer, epithelium, and the underlying lamina propria. These create a physical barrier to gut microbes using multi-protein complexes called tight junctions. When the tight junctions are compromised, epithelium permeability increases, causing a leaky gut. A leaky gut is responsible for the development of many gastrointestinal conditions, such as irritable bowel syndrome, irritable bowel disease, and celiac disease. Probiotics can upregulate ZO-1 and occludin protein synthesis, thus protecting the integrity of the gut barrier [35].
**(iii)**  **Producing antimicrobial substances:** Probiotics produce a variety of substances that can be inhibitory to both gram-positive and gram-negative bacteria. These substances include hydrogen peroxide, bacteriocins, and organic acids. This can not only reduce the number of pathogenic bacteria but can also alter bacterial metabolism and limit toxin production [36,37].
**(iv)**  **Modulating the gut microbiome:** Probiotic use has been shown to re-equilibrate gut microbiome dysbiosis. Dysbiosis can occur when a patient is exposed to severe conditions such as prolonged antibiotic therapy, intense physical stress, and chronic illness. Probiotics metabolize complex carbohydrates and produce lactic acid and short-chain fatty acids. This reduces bacterial translocation, improves tight junction integrity, and stimulates mucin production [38].
**(v)**  **Increasing water absorption:** Aquaporins are water-channel membrane proteins expressed in many tissues with AQP1, 3, 4, and 8 mostly expressed in the colon. Pathogenic bacteria can disrupt these proteins, increase the water content in stool, and lead to dehydration. Probiotics have been shown to increase the expression of aquaporins and thus increase water absorption in the colon [39].
**(vi)**  **Decreasing opportunistic pathogens:** Probiotics decrease the number of pathogenic bacteria by producing inhibitory substances such as bacteriocins, blocking adhesion sites on the intestinal epithelial surfaces, and competing for nutrients. These mechanisms are important for prophylaxis and the treatment of infections. The ability of probiotics to co-aggregate can lead to a protective barrier that prevents pathogenic bacteria to colonize the epithelium [40].

## 6. Randomized Controlled Trials (RCT)

Past studies have shown that probiotics are useful in the prevention of AAD [41]. However, many of the previous reviews were focused on the inpatient setting, which differs in the intensity of antibiotic treatment, the route of drug administration, and the potential pathogens involved. There have since been systematic reviews and meta-analyses to assess the benefits and disadvantages of probiotics in preventing AAD for all ages in the outpatient setting as well. In one such meta-analysis, Blaabjerg et al. identified 17 prospective, randomized controlled trials with placebo, active, or no-treatment control arms in testing probiotics for the prevention of AAD in an outpatient setting [42]. It was observed that probiotics conferred a protective effect to prevent AAD in the outpatient setting for all ages. From a total of 3631 patients in the study, probiotics reduced the risk of AAD by 51% (RR 0.49; 95% CI 0.36 to 0.66; I^2^ = 58%), with no apparent increase in the risk of side effects. In total, the number needed to treat (NNT) to prevent one case of diarrhea was 11 (95% CI 6 to 13). However, bias, definitions of diarrhea, types of infections, and the type of antibiotic used varied, which should be a consideration in the interpretation of results.

The data from meta-analysis and RCTs are only as useful as their clinical correlation. Identifying an appropriate probiotic product from the vast amount of data can be a daunting task. Expert consensus and current guidelines recommend that reviews and meta-analyses show outcome data through probiotic strain sub-groups to truly assess efficacy outcomes and help clinicians best select treatment options [43,44,45]. However, direct comparisons of strains are rare and commonly the strain is tested for the same indication. By assessing and synthesizing data from RCTs by strain, clinicians can better target their treatments (Table 1).

## 7. Probiotics for Reducing *C. difficile*-Associated Diarrhea

*C. difficile* is the leading cause of both hospital- and community-acquired AAD [60]. As a growing health problem across the world, *C. difficile* infection is associated with significant healthcare costs and rising morbidity and mortality. *C. difficile*-associated diarrhea (CDAD) occurs more commonly in the elderly patient population and hospitalized patients receiving broad-spectrum antibiotics. One of the most significant risk factors for *C. difficile* infection is antibiotic exposure, and certain drugs such as cephalosporins, penicillin, clindamycin, and fluoroquinolones are implicated in *C. difficile*-associated diarrhea. Studies for the prevention of *C. difficile* infection mainly focus on limiting the spread. This includes early isolation, proper hand hygiene, personal protective equipment, and environmental cleaning. However, more recently, probiotics have been proposed for the prevention and treatment of *C. difficile*-associated diarrhea. Probiotic supplementation has been shown to significantly reduce the risk of developing *C. difficile*-associated diarrhea in patients receiving antibiotics, especially hospitalized patients, as these reduce colonization by *C. difficile* [61,62,63,64,65,66].

## 8. Considerations for Probiotics Use

As more data is released on the benefits of probiotics to combat gastrointestinal diseases such as AAD, physicians are better able to tailor prevention strategies to their patient populations. Probiotics can be found in the form of yogurt, kefir, kombucha, tablets, capsules, etc. Probiotics have become quite accessible to the public, as one can find a source in most supermarkets, pharmacies, and supplement stores. However, the actual definition of probiotics has fallen victim to commercial marketing strategies and thus become diluted. In 2014, a refined yet similar definition of probiotics came out of a consensus panel that defined them as “Live microorganisms that when administered in adequate amounts confer a health benefit on the host” [67]. Within the clinical sector, it is imperative that we collectively define a strict guideline for probiotics that can first be established through research. Three key elements have been identified to determine the validity of a probiotic and help narrow the scope [68]. This includes evidence that the strain has been tested in an RCT in a heterogeneous or stratified population, dosing is equal to the human trial, and the whole genome is characterized and transparent. Using guidelines can help focus therapies on specific patient populations, limit adverse events, and improve therapeutic outcomes.

## 9. Safety of Probiotics

Probiotics have been used safely for years. Meta-analysis has found few increased risks of adverse effects [69]. Some adverse events that are reported in RCTs studying the use of probiotics in the prevention of AAD include abdominal pain, nausea, loss of appetite, headache, and flu-like symptoms. However, the symptoms are most likely due to antibiotic side effects or from an underlying infection. Data emerging from clinical cases and trials, and experimental models have demonstrated potential risks [70]. It is important to keep in mind that probiotic strains differ in the clinical effects they exert and also may have different safety profiles. The probiotic organism used in the commercial preparations, as well as the other components of that product, influences its safe use. Probiotics can be responsible for systemic infections, bowel ischemia, excessive inflammation, gene transfer between microbiome bacteria and probiotics, and gastrointestinal side effects such as cramping and nausea. Although these findings are rare, it is recommended that investigators who carry out clinical trials using probiotics conduct active surveillance for adverse effects and identify any patients who are at higher risk [71,72,73,74,75,76,77,78].

## 10. Practicality and the Current Clinical Practice of Using Probiotics in AAD

At present, there is no consensus across the globe regarding the recommendation for the clinical use of probiotics for AAD. This emphasizes the urgent need for further research and the effective distribution of information as it emerges. A global panel of experts (World Gastroenterology Organization; WGO) reviewed the literature and made several evidence-based recommendations for the use of probiotics for various disease conditions. They suggested that there is evidence to support that certain probiotics are effective for the prevention of AAD in adult and pediatric patient populations [79]. The Canadian Agency for Drugs and Technologies in Health (CADTH) carried out an evaluation of probiotics for AAD in the pediatric population [80]. A strong recommendation was made for the use of *L rhamnosus GG* and *S. boulardii* for preventing AAD in children, while the use of *B. clausii* as a single probiotic was not recommended [81]. The Canadian Paediatric Society recommended that physicians should consider advising the use of probiotics for AAD, but should be aware of the small risks of invasive infections with certain strains, especially while treating immunocompromised patients. They also recommended that the federal government should require probiotics manufacturers to provide accurate and detailed labels and maintain the high quality of the products [82].

As the common form of infectious diarrhea related to the use of antibiotics is caused by *C. difficile*, several clinical guidelines for the use of probiotics are discussed with respect to *C. difficile* infection. The guidelines for the use of probiotics for the treatment of AAD, including that associated with a *C. difficile* infection, are varied across the world. For example, the American College of Gastroenterology (ACG) guidelines recommended against the use of probiotics for the prevention of both primary and recurrent infections of *C. difficile* [83]. On the other hand, the American Gastroenterological Association (AGA) suggested the use of certain organisms such as *S. boulardii*, or combinations of strains, for example, *L. acidophilus* CL1285 and *L. casei,* for adults and children who are being treated with antibiotics. They also cautioned against the use of probiotics in patients with severe illnesses or those who are concerned about the cost of the probiotics [84]. It was argued that the difference between these two guidelines may be due to the number of randomized trials considered for making the respective recommendations and also differences in the strain specificity taken into account by the individual agencies [85]. However, it was agreed that more research is needed [86]. The European Society of Clinical Microbiology and Infection (ESCMID) guidelines do not recommend probiotics to be used as an adjunctive *C. difficile* treatment [87]. Consistent with this recommendation, the Infectious Diseases Society of America (IDSA) together with the Society for Healthcare Epidemiology of America (SHEA) also put forth similar guidelines. These agencies cited the lack of evidence for (i) probiotics being beneficial and (ii) completely without harm as reasons for their recommendations [88].

## 11. Conclusions

Research on microbial administration to modulate the human microbiome and improve health has been increasing at a rapid rate since probiotics were officially defined. The potential to alter these microbial ecosystems offers great hope for new preventative treatment options for antibiotic-associated diarrhea and other gastrointestinal disorders. However, the practical guidelines for probiotic use that provide strain-specific and disease-specific recommendations are sparse. Several studies show benefits of the use of probiotics for AAD, but still call for large placebo-controlled trials (i) to determine species and dose effectiveness for prevention, (ii) to show effectiveness in preventing AAD, and (iii) to establish the effect on length of hospital stay and cost-effectiveness [89,90,91]. Going forward, research should focus on strain-specific, dosing requirements, safety concerns, and patient factors that can help clinicians make targeted choices on probiotic use for treating antibiotic-associated diarrhea and other gastrointestinal disorders.

## Figures and Tables

**Table 1:** Randomized controlled trials of probiotic use in the prevention of AAD.

Probiotic Genus and Strain	Outcome of RCTs
*Lactobacillus acidophilus*	It significantly shortened the duration of diarrhea [46] and improved gastrointestinal complaints [47].The probiotic preparation did not consistently prevent amoxicillin-induced diarrhea in a pediatric population [48] or reduce the side effects of *H. pylori* triple therapy [49].
*Lactobacillus rhamnosus GG*	*Lactobacillus GG* overall significantly reduced stool frequency and increased stool consistency during antibiotic therapy by the tenth day compared with the placebo group [50]. The absolute risk reduction of diarrhea was 11% within two weeks of antibiotic therapy [51]. Patients using this probiotic showed improvements with respect to the number of bowel movements (*p* < 0.10) and feces consistency ratings by the investigators (*p* < 0.05) at the study end [52].
*Lactobacillus reuteri*	*L. reuteri* significantly reduced the diarrhea rate by 24 h during antibiotic use. It also increased the eradication rate in *H. pylori* patients and decreased the occurrence of the most common side effects that are observed with antibiotic treatment [53].
*Bacillus subtilis* and *Streptococcus faecium*	Supplementation with probiotic strains, composed of *Bacillus subtilis* and *Streptococcus faecium*, was shown to improve drug compliance, reduce side effects, and enhance the intention-to-treat eradication rate of *H. pylori* [54].
*Saccharomyces boulardii*	*S. boulardii* decreased the diarrhea rate from 32.3% to 11.4% in a pediatric group receiving sulbactam-ampicillin [55], prevented AAD and decreased side effects when given with *H. pylori* treatments [56,57,58,59], and significantly reduced the rate of AAD when given prophylactically with a beta-lactam antibiotic [59].
